# Editorial: Stroke and balance disorders

**DOI:** 10.3389/fstro.2025.1560735

**Published:** 2025-02-18

**Authors:** Arlindo C. Lima Neto, Michael Strupp, Alexander A. Tarnutzer, Andreas Zwergal, Emilio Domínguez-Durán, Camila Giacomo Carneiro

**Affiliations:** ^1^Department of Otorhinolaryngology, Clinics Hospital of University of São Paulo, São Paulo, Brazil; ^2^Department of Neurology & German Center for Vertigo and Balance Disorders, Ludwig Maximilians University Hospital, Ludwig Maximilians University Munich, Munich, Germany; ^3^Cantonal Hospital of Baden, Baden and University of Zurich, Zurich, Switzerland; ^4^Hospital Quiron Salud Infanta Luisa, Seville, Spain; ^5^Department of Otorhinolaryngology, Faculty of Medicine of Ribeirao Preto, University of Sao Paulo, Ribeirão Preto, Brazil

**Keywords:** vascular, vertigo, dizziness, stroke, TIA, emergency department (ED), HINTS battery, benign paroxysmal positional vertigo (BPPV)

Despite significant advancements in the understanding of Vascular Vertigo and Dizziness (VVD), including new diagnostic criteria and guidelines, significant gaps remain in the pathophysiology, etiology, clinical presentation, and management of these patients (Kim et al., 2022; Edlow et al., 2023). This is partly due to the broad range of clinical presentations of patients with vertebrobasilar (VB) stroke, involving different parts of the brainstem and cerebellum, including overlapping central and peripheral lesions, such as the anterior inferior cerebellar artery (AICA) infarction, resulting in vestibular and audiologic symptoms (Kim et al., 2022) (Figure 1).

**Figure 1:**
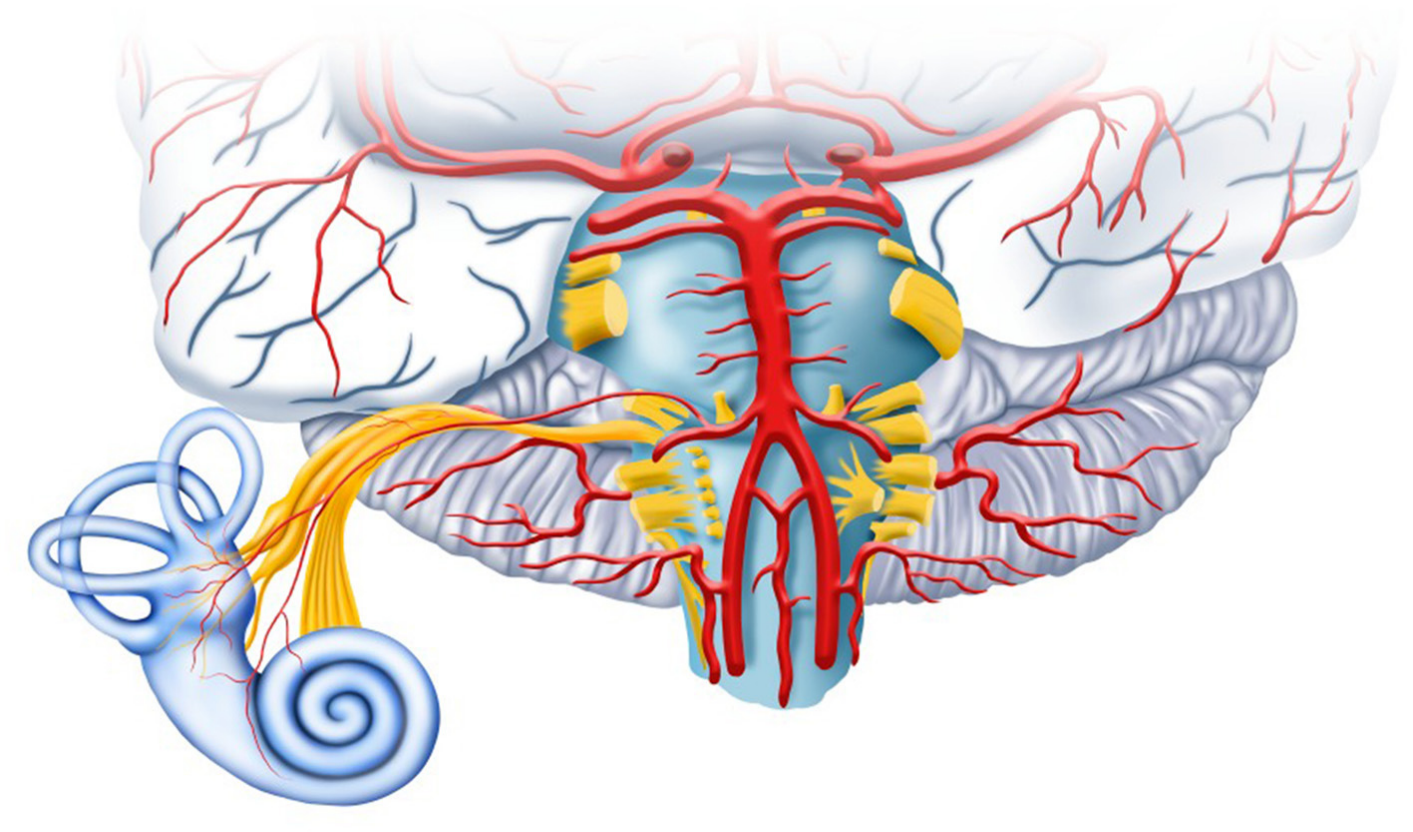
The vertebrobasilar circulation provides blood supply to the cerebellum, the brainstem, and the inner ear.

The current classification of VVD includes vertigo/dizziness due to stroke or VB transient ischemic attack (VBTIA), labyrinthine infarction/hemorrhage, and vertebral artery compression syndrome (Kim et al., 2022). Consequently, much research on this area has focused on emergency department (ED) management, emphasizing the differentiation from other causes of acute, episodic, and persisting dizziness, which is crucial for accurate diagnosis and timely intervention.

Among many diagnostic algorithms for acute VVD, the HINTS (Head Impulse, Nystagmus, and Test of Skew), HINTS+ (plus testing of hearing), and STANDING are cornerstones (Tarnutzer and Edlow, 2023). However, many challenges remain, and further refinement is necessary. In this Research Topic, Morrow et al. highlighted that the crucial phase in the diagnostic process is the “hyperacute” period—the initial hours following symptom onset—which determines triage and treatment decisions. Despite its importance, none of the established algorithms have been validated for use during this phase. Their review emphasizes the timing of diagnostic tests within the ED pathway, resource availability, and the pitfalls associated with current practices (Kim et al., 2022; Edlow et al., 2023; Tarnutzer and Edlow, 2023; Filippopulos et al., 2022).

The take-up of these diagnostic algorithms is limited because many emergency physicians are unfamiliar with them. Bierrum et al. discussed the potential benefits of establishing an acute vertigo service within the ED, including economic advantages, reduction of unnecessary investigations, and shorter hospital stays. Otherwise, it's necessary to properly train frontline emergency physicians.

In this regard, Ursat et al. evaluated the use of a mannequin-based virtual reality simulator to train clinicians in the HINTS protocol. Their study showed improved diagnostic performance in the trained group compared to a control group that only received theoretical lessons and video demonstrations (89% sensitivity and 100% specificity vs. 45% and 86%, respectively, *p* < 0.001).

Furthermore, training in the recognition of positional nystagmus is essential. Koohi, Male, et al. evaluated diagnoses made by emergency physicians vs. acute vertigo specialists. Of 71 patients, clinicians identified 13 patients with peripheral paroxysmal positional vertigo (BPPV) and no central positional nystagmus, while specialists diagnosed 9 patients with BPPV and 6 with central positional nystagmus.

In addition to the head impulse test, assessing positional and spontaneous nystagmus (SN) is essential for bedside testing in the ED (Edlow et al., 2023; Filippopulos et al., 2022). Wüthrich et al. conducted a systematic review of SN patterns from 39 studies, revealing that horizontal or horizontal-torsional SN is more common in peripheral etiologies (94.8% vs.43.4%, *p* < 0.001), while torsional or vertical SN patterns are observed significantly more frequently in central disorders (15.1% vs. 2.6%, *p* < 0.001).

The HINTS+ test, while valuable, has limitations, particularly in the absence of SN during patient evaluation (Tarnutzer and Edlow, 2023). To address this, Fracica et al. explored additional tools, such as smooth pursuit and saccadic eye movements, focusing on anterior and posterior circulation strokes. These tools may reveal highly localized findings that can distinguish between peripheral and central etiologies in some cases.

Out-of-pattern symptoms may occur due to the anatomical complexity of the vasculature and pathways of the posterior fossa and inner ear (Figure 1). For example, Koohi, Haider, et al. described a case of contralateral (right) sudden-onset sensorineural hearing loss with isolated acute central vestibular dysfunction due to a left pontine infarction. The authors explain that fibers from the contralateral auditory nucleus in the medulla pass through the pons and midbrain and terminate in the temporal lobe. Dysfunction of the crossed lateral lemniscus pathways leads to unilateral hearing loss.

Another rare condition presenting as acute vertigo is isolated (hemi)nodular infarction. Lee et al. reported on a case series of six patients with a range of clinical symptoms, including horizontal SN (*n* = 2), positional nystagmus (5/6), head-shaking nystagmus (3/6), oblique gaze deviation (*n* = 1), and truncal ataxia (5/6). Despite these symptoms, vestibulo-ocular reflex (VOR) gain, measured by video head impulse testing, was normal in all cases, confirming the preservation of the VOR in (hemi)nodular strokes.

Beyond the ED, recurrent VVD episodes, particularly those resulting from VBTIAs, require attention. Lima Neto et al. investigated a cohort of 103 patients with recurrent VBTIAs and found that secondary prophylaxis (aspirin, clopidogrel, aspirin plus clopidogrel, and rivaroxaban) reduced recurrence frequency by 93.2% compared with the previous period of no medication, within a median follow-up of 12 months (range = 2 to 36 months). Only 7 patients experienced a new attack while on medication, but no prognostic factors for recurrence were identified.

In patients with stroke sequelae, the risk of falling is often associated with a heightened fear of falling. Addressing this cycle through comprehensive treatment strategies can improve functional outcomes, reduce anxiety, and enhance quality of life (Xu et al., 2018). Abdelfadil et al. in a sample of 40 stroke patients, demonstrated that a protocol combining systematic desensitization (a cognitive-behavioral therapy) with a goal-directed paradigm improves functional performance (measured by Timed Up and Go test and Dynamic Gait Index) and reduces both the risk and fear of falling (accessed by the Berg Balance Scale, the Biodex Fall Risk Index, and the Fall Efficacy Scale-International) in comparison to a goal-directed paradigm alone (*p* < 0.001 for all variables).

The goal of this Research Topic is to advance scientific understanding of the prevention, recognition, and management of VVD. With the Research Topic of articles included we cover the impact of triage and diagnostic work-up strategies (Morrow et al., Bierrum et al.), bedside diagnostic algorithms (Wüthrich et al., Fracica et al., Koohi, Male, et al.), dedicated exercise (Ursat et al.), and quantitative vestibular testing (Lee et al.). Furthermore, secondary prevention strategies in patients with VBTIAs (Lima Neto et al.) and potential treatment approaches to fear of falling in stroke patients (Abdelfadil et al.) are also addressed. Thus, the 10 articles of this Research Topic provide a comprehensive and up-to-date resource for both researchers and clinicians.

## References

[B1] Edlow J. A. Carpenter C. Akhter M. Khoujah D. Marcolini E. Meurer W. J.. (2023). Guidelines for reasonable and appropriate care in the emergency department 3 (GRACE-3): acute dizziness and vertigo in the emergency department. Acad. Emer. Med. 30, 442–486. 10.1111/acem.14728 37166022

[B2] Filippopulos F. M. Strobl R. Belanovic B. Dunker K. Grill E. Brandt T.. (2022). Validation of a comprehensive diagnostic algorithm for patients with acute vertigo and dizziness. Eur. J. Neurol. 29, 3092–3101. 10.1111/ene.15448 35708513

[B3] Kim J. S. Newman-Toker D. E. Kerber K. A. Jahn K. Bertholon P. Waterson J.. (2022). Vascular vertigo and dizziness: diagnostic criteria. J. Vestib. Res. 32, 205–222. 10.3233/VES-210169 35367974 PMC9249306

[B4] Tarnutzer A. A. Edlow J. A. (2023). Bedside testing in acute vestibular syndrome-evaluating HINTS plus and beyond-a critical review. Audiol. Res. 13, 670–685. 10.3390/audiolres13050059 37736940 PMC10514811

[B5] Xu T. Clemson L. O'Loughlin K. Lannin N. A. Dean C. Koh G. (2018). Risk factors for falls in community stroke survivors: a systematic review and meta-analysis. Arch. Phys. Med. Rehabil. 99, 563–573.e565. 10.1016/j.apmr.2017.06.032 28797618

